# Transcriptome dynamics of *Arabidopsis thaliana* root penetration by the oomycete pathogen *Phytophthora parasitica*

**DOI:** 10.1186/1471-2164-15-538

**Published:** 2014-06-29

**Authors:** Agnès Attard, Edouard Evangelisti, Naïma Kebdani-Minet, Franck Panabières, Emeline Deleury, Cindy Maggio, Michel Ponchet, Mathieu Gourgues

**Affiliations:** UMR Institut Sophia Agrobiotech, INRA1355-CNRS7254-UNSA, Université de Nice Sophia-Antipolis, 400 route des chappes, F-06903 Sophia Antipolis, France; Sainsbury Laboratory (SLCU), University of Cambridge, Bateman Street, Cambridge, CB2 1LR UK

**Keywords:** *Phytophthora*, *Arabidopsis thaliana*, Appressorium, Transcriptome, Root infection

## Abstract

**Background:**

Oomycetes are a group of filamentous microorganisms that includes both animal and plant pathogens and causes major agricultural losses. *Phytophthora* species can infect most crops and plants from natural ecosystems. Despite their tremendous economic and ecologic importance, few effective methods exist for limiting the damage caused by these species. New solutions are required, and their development will require improvements in our understanding of the molecular events governing infection by these pathogens. In this study, we characterized the genetic program activated during penetration of the plant by the soil-borne pathogen *Phytophthora parasitica*.

**Results:**

Using all the *P. parasitica* sequences available in public databases, we generated a custom oligo-array and performed a transcriptomic analysis of the early events of *Arabidopsis thaliana* infection. We characterized biological stages, ranging from the appressorium-mediated penetration of the pathogen into the roots to the occurrence of first dead cells in the plant. We identified a series of sequences that were transiently modulated during host penetration. Surprisingly, we observed an overall down regulation of genes encoding proteins involved in lipid and sugar metabolism, and an upregulation of functions controlling the transport of amino acids. We also showed that different groups of genes were expressed by *P. parasitica* during host penetration and the subsequent necrotrophic phase. Differential expression patterns were particularly marked for cell wall-degrading enzymes and other proteins involved in pathogenicity, including RXLR effectors. By transforming *P. parasitica* with a transcriptional fusion with GFP, we showed that an RXLR-ecoding gene was expressed in the appressorium and infectious hyphae during infection of the first plant cell.

**Conclusion:**

We have characterized the genetic program activated during the initial invasion of plant cells by *P. parasitica*. We showed that a specific set of proteins, including effectors, was mobilized for penetration and to facilitate infection. Our detection of the expression of an RXLR encoding gene by the appressorium and infection hyphae highlights a role of this structure in the manipulation of the host cells.

**Electronic supplementary material:**

The online version of this article (doi:10.1186/1471-2164-15-538) contains supplementary material, which is available to authorized users.

## Background

Plant pathogenic oomycetes have a particular physiology and are known for their devastating effects on agricultural crops and natural ecosystems. A small number of oomycetes, including downy mildews and *Phytophthora* and *Pythium* species, which are pathogenic on virtually all dicots and on some cereals [1], have a major impact on agriculture worldwide. Oomycete control strategies are currently very limited, because very few chemicals are effective against these microorganisms. Indeed, most of the molecules used to reduce the incidence of plant diseases caused by filamentous pathogens target fungal functions that are absent or dispensable in oomycetes, such as melanin, sterol or chitin biosynthesis. These organisms are phylogenetically related to brown algae and diatoms within the Stramenopiles and they have unusual biological, genetic and physiological features [2, 3]. The selection of resistant plant genotypes remains an efficient anti-oomycete strategy, but this approach is costly and time-consuming and is, thus, restricted to crops with a high added value. The development of new methods to combat oomycetes thus requires improvements in our knowledge of the physiology and infection strategies of these pathogens. In particular, early events in infection, including the mechanisms underlying penetration and the modulation of plant responses to ensure successful infection are poorly documented. The modification of these processes through the development of new chemicals or plant engineering would reduce the incidence of diseases caused by oomycetes.

Characterizations of plant penetration by oomycetes at the cellular level have focused mostly on *Phytophthora* spp. Entry into the host is mediated principally by a specialized cellular structure called the appressorium [4–8]. Poor nutrient content, surface hydrophobicity and topography have been shown to induce appressorium differentiation in *Phytophthora infestans* [9]. This process also requires calcium [10]. Little is known about the molecular events governing the differentiation and functioning of appressoria. Gene inactivation strategies have clearly demonstrated a requirement for four proteins for the penetration process. The RNAi-mediated silencing of a family of four cellulose synthase genes from *P. infestans* revealed that cell wall organization is important for appressorium differentiation and plant infection [11]. The *PiHMP1* gene encodes a membrane protein that accumulates in appressoria and haustoria and is required for early infection [12]. The PiBZP1 transcription factor from *P. infestans* is required for appressorium differentiation [13]. Finally, the silencing of the *P. sojae* mitogen-activated protein kinase PsSAK1 greatly decreases appressorium differentiation, providing a first clue to the signaling pathways involved in the control of this process [14].

Beyond candidate gene silencing strategies, a few medium- to large-scale analyses have been performed to decipher the molecular mechanisms governing appressorium differentiation in *Phytophthora* spp*.* Kramer and coworkers first detected, by 2D-SDS-gel electrophoresis, stage-specific peptides in *P. infestans* appressorium-like structures differentiated on artificial surfaces [15]. A comparative analysis then showed an accumulation of transcripts and proteins involved in amino-acid synthesis during the formation of appressorium-like structures *in vitro* [16]. These findings were confirmed by a second analysis of protein extracts from appressorium-like structures [17]. This study highlighted the accumulation of proteins involved in protein synthesis and energy metabolism, together with putative pathogenicity factors, such as the Crinkling and Necrosis protein CRN2 and proteins involved in protection against reactive oxygen species. Grenville-Briggs and coworkers recently made use of a coupled liquid chromatography/MS-MS system to identify membrane and cell wall-associated proteins [18]. These proteins included a transglutaminase, a glycosyl hydrolase and a group of three previously unknown but related proteins containing two repeats that accumulated in *P. infestans* appressoria. Numerous proteins previously characterized as PAMPs (pathogen-associated molecular patterns), such as CBEL-like proteins or elicitins, and crinkler-like proteins accumulated in the cell walls of germinating cysts, suggesting that all these proteins accumulate before infection. Recent transcriptome studies, based on full genome data for *P. infestans, P. sojae* and *P. capsici*, have highlighted the upregulation of transcripts encoding proteins involved in gene expression and translation, primary metabolism, protein kinases, cell wall-degrading enzymes (CWDE) and various proteins used to manipulate plant cells (effectors) during *in vitro* appressorium differentiation [19–21]. Similar observations have been obtained with an expressed sequence tag (EST) approach applied to the broad-host range pathogen *P. parasitica*. An investigation of the genes expressed during appressorium formation identified sequences encoding numerous cell wall-degrading enzymes and pathogenicity-related proteins [8].

Taken together, these analyses provide interesting clues to the developmental program occurring during appressorium differentiation. However, all the studies investigating early events in plant-oomycete interactions have made use of artificial surfaces to induce appressorium differentiation or were performed on aerial parts of plants [16, 18–20]. Artificial hydrophobic surfaces cannot be pierced and only the events from zoospore encystment to the differentiation of appressorium-like structures can be analyzed. It is not possible to characterize the steps from appressorium maturation to early plant penetration with such systems. Furthermore, most pathogenic oomycetes infect plant roots and the molecular events described on aerial tissues may not reflect those governing root infection. Information is therefore required concerning the molecular events occurring during the appressorium-mediated penetration of the host root system by oomycetes.

We addressed this question, by analyzing early events in *P. parasitica* infection by a transcriptome analysis. This species infects the roots of a wide range of plants and is emerging as a model species [22]. We made use of the *P. parasitica/Arabidopsis thaliana* pathosystem to analyze the events occurring during the first few hours after the inoculation of roots with motile zoospores [23]. We hybridized a custom oligoarray containing almost one quarter of the *P. parasitica* genome with samples recovered from a time-course of infection ranging from penetration to the switch to necrotrophy. We identified a subset of sequences that specifically accumulated or were repressed during appressorium-mediated penetration of the host. We then investigated the functions of the proteins encoded by these sequences.

## Methods

### *P. parasitica*and plant culture conditions

*Phytophthora parasitica* INRA-310 strain, selected as the reference for the *P. parasitica* genome sequencing project was mainly used for this study. Cultures were performed on V8 Medium and zoospore production was induced as previously described [24]. A *P. parasitica* race 0 strain was kindly provided by Elodie Gaulin (Toulouse III University) and was used for transformation experiments.

*Arabidopsis thaliana* plantlets were grown, inoculated and observed as previously described [23]. *A. thaliana* Col-O ecotype was used. An *A. thaliana* transgenic line expressing the mCherry plasma membrane marker PM-RB was kindly supplied by Professor Torii from Washington University and was used for the cytological analysis of the expression profile of a *P. parasitica* appressorium specific gene [25].

Appressorium differentiation was induced on onion epidermis as previously described [8].

### Sample preparation and RNA extractions

For hybridization and subsequent validations of expression patterns by RT-qPCR, a series of two biological replicates each corresponding to RNA extractions of the following biological conditions were used: 1-Vegetative mycelium (recovered from two samples of 4 day-old cultures in liquid V8 medium at 24°C), 2- Motile zoospores (recovered from 8 independent cultures), 3-Appressoria differentiated on onion epidermis (epidermis from 20 onion bulbs inoculated with zoospores collected from 8 independent Petri dishes); appressoria collected 3 hours after inoculation (24°C), 4- Infection of *A. thaliana* roots by *P. parasitica* zoospores (samples recovered at 2.5, 6, 10.5, 30 and 96 hours post inoculation; 5 inoculated plants for each sample) as already reported [23].

As a rule, independent samples were used for Array hybridizations and RT-qPCR analyzes with an exception for purified appressoria RNA that are hardly obtained. RNA extraction from inoculated plant tissues was performed as described by [26]. *P. parasitica* RNA was extracted using Trizol reagent (Invitrogen, France).

### *P. parasitica*oligoarray design and hybridizations

An unisequence set was obtained by clustering all *P. parasitica* EST sequences available in dbEST (Additional file 1: Figure S1). They originated from vegetative mycelium [27], zoospores and germinating cysts [28, 29], appressoria [8] and *P. parasitica*-infected tomato plantlets, displaying symptoms of the necrotic step of the invasion [30]. Clustering was performed using TGICL package (http://compbio.dfci.harvard.edu/tgi/software/) and default parameters (Additional file 1: Figure S1). Unisequence composition is detailed in the supporting information (Additional file 2: Table S1). Ascribing sequences to plant or *Phytophthora* was initially done as already described [8]. This was supported by a subsequent BLASTN search against the recently released *P. parasitica* genome V2.0 (http://www.broadinstitute.org). Functional annotation was performed using blastx against the NCBI non redundant protein database (E value < 1E-05), searches against the Interpro database [31] and using Blast2GO annotation tool [32]. The *P. parasitica* oligoarray manufactured by NimbleGen systems (NimbleGen Systems, Reykjavik, Iceland) contained 11 independent 60-mer probes per unisequence with 4 technical replicates (Additional file 1: Figure S1). This array is fully described in the platform GPL17781 stored in the Gene Expression Omnibus (GEO) at NCBI (http://www.ncbi.nlm.nih.gov/geo).

CDNA synthesis, sample labeling, hybridization procedures, data acquisition and normalization were performed at the NimbleGen facilities (NimbleGen Systems, Reykjavik, Iceland). The complete expression dataset is available as series accession number GSE51252 in the GEO at NCBI. Average expression levels were calculated for each gene from the independent probes on array and were used for further analysis. Genes were considered as not expressed in a sample (background level) if the normalized value was less that the 95^th^ percentile of random probes found on the array. Data were subjected to the Anaïs statistical framework to identify differentially expressed sequences (Pvalue <0.05) [33]. Data were mean-centered and log-2 transformed using Epclust (http://www.bioinf.ebc.ee/EP/EP/EPCLUST/). Hierarchical clustering (Pierson correlations, average linkage) and K-mean clustering (default parameters) were performed using Genesis program [34].

### Real time RT-PCR analyses

Total RNA was treated with DNAse I (Ambion, Austin, USA) and reverse transcribed (1 μg) to cDNA using I-Script cDNA synthesis (Biorad, Hercules, USA). Real Time PCR experiments were performed using 5 μl of 1:50 dilution of first strand cDNA and SYBRGreen (Eurogentec SA, Seraing, Belgium) using the Opticon 3 (Biorad, Hercules, USA). All assays were carried out in triplicates. Gene-specific oligonucleotides were designed using primer3 (http://frodo.wi.mit.edu) and their specificity was validated by the analysis of dissociation curves using genomic DNA as a template. The genes encoding ubiquitin conjugating enzyme (Ubc, CK859493), the 40S ribosomal protein S3A (WS21, CF891675), and the *P. parasitica* homolog (PpGAM26e01: BlastN, 91% identity) of the *P. infestans* Mago-Nashi protein (Pi000681) described to be stably expressed were selected as constitutive internal controls [19, 35]. Quantification of gene expression was performed using delta CT method [36].

### GO enrichment analysis

Full Gene Ontology files were downloaded at http://www.geneontology.org/GO.downloads.ontology.shtml. After reconstitution of GO pathways, occurrences of each GO term and their parent terms were numbered for each cluster and for the *P. parasitica* array dataset. Proportion of GO terms in each cluster was then compared to the proportion observed on the *P. parasitica* array. Terms with significant enrichment were identified using Fisher Exacts test with a p-value cut off at 0.05. To facilitate the analysis, only GO parent terms with level higher than 4 were considered.

### *P. parasitica*transformation

A Gateway vector was obtained to accelerate gene expression analyses in *Phytophthora* spp. using transcriptional and translational fusions. The reporter system, corresponding to a fusion between Green Fluorescent protein (GFP) and Escherichia coli β-glucuronidase (GUS), was derived from the pKGWSF7 vector dedicated to plant transformation (from University of Gent, http://gateway.psb.ugent.be/; [37]). It was modified to be suitable for *Phytophthora* transformation. A geneticin resistance cassette was obtained as a ClaI (blunt ends with DNA pol I)/ SacI fragment from the pTefGHNH vector [30]. It contains the *P. parasitica* translation elongation factor 1 (*Tef1*) promoter, the NPTII coding sequence conferring geneticin resistance and the Ham34 terminator from *Bremia lactucae*. This fragment was cloned into a vector fragment corresponding to an AflII (blunt)/SacI fragment from the pKGWFS7 Gateway cloning vector, replacing the kanamycin resistance cassette used for plant selection. The resulting plasmid was named pIPO-1.

To obtain a translational fusion between the promoter sequence of a RXLR encoding gene (CL380) with the GFP-GUS reporter system (pCL380::GFP-GUS), a 1144 bp fragment upstream of CL380 sequence start codon was amplified on genomic DNA from *P. parasitica* PP-INRA310 and cloned into the pIPO-1 vector. *P. parasitica* race 0 protoplast transformation was performed as previously described [30].

### Confocal microscopy

A Zeiss LSM 510 META confocal microscope was used (Carl Zeiss GmbH, Jena, Germany). GFP excitation was obtained at 488 nm. Penetration structures were observed two to thirty hours after inoculation of 5×10^5^ zoospores on onion epidermis. Infection of *A. thaliana* roots was observed eight and thirty hours after inoculation of 5×10^5^ zoospores. For imaging of penetration and invasion on onion epidermis, acquisition parameters were calibrated at two hours post inoculation and unchanged at the following time points to enable comparison.

## Results

### Design of the *P. parasitica*oligoarray

We retrieved all the *P. parasitica* EST sequences available from public databases. These sequences were obtained at various developmental stages and from cell types involved in plant infection: mycelium, zoospores, appressoria differentiated on onion and tomato necrotrophic stage of infection [8, 27–30]. In total, 12632 ESTs were assembled into 1572 contigs and 3879 singletons, giving a final 5451-unisequence set (Additional file 2: Table S1). A NimbleGen custom oligo-array was designed (Additional file 1: Figure S1). Approximately 97% of the unisequences were represented by more than five 60-bp probes. Only 24 sequences did not fit the NimbleGen criteria for probe design and were discarded from the analysis. Following this design step, 56524 probes corresponding to 5427 sequences were spotted onto the array. We attributed 4398 sequences (81%) to *P. parasitica* and 729 sequences (13%) to the plant (Additional file 1: Figure S1). The origin of 324 sequences (6%) remained unknown (Additional file 3: Table S2). In total, 4700 probe sets corresponded to sequences from *P. parasitica* or of unknown origin. The remaining probe sets matched sequences of plant origin. Finally, 163000 random probes were added to complete the array, as controls.

Blastx searches of sequences attributed to *P. parasitica* against a protein set based on the recently released genome annotation gave 3806 hits with identity levels of more than 95%. Blastx searches (e-value = 1E-20) using the 4398 *P. parasitica* sequences as queries also revealed that 320 sequences (7%) had no ortholog in the genomes of *P. infestans*, *P. ramorum and P. sojae* and therefore constituted probable species-specific sequences. Functional annotation of the 4398 and 324 sequences attributed to *P. parasitica* and of unknown origin, respectively, was performed following blastx searches (E-value cut off: 1E-05) against the NCBI non-redundant (NR) protein database, with the blast2GO tool [32]. A GO term was associated with 3516 sequences (65%), whereas the function of 1935 sequences remained unknown (Additional file 3: Table S2).

### Analysis of gene expression during early steps of the *P. parasitica*/*A. thaliana*interaction

The custom oligo-array was used to identify the modulations of the *P. parasitica* transcriptome during the onset of a compatible interaction. The samples analyzed corresponded to vegetative mycelium, motile zoospores, purified appressoria and a time course of early infection in *A. thaliana*. The *A. thaliana* samples were collected 2.5, 6, 10.5 and 30 hours after inoculation (hai), from roots inoculated with *P. parasitica* motile zoospores, thus reflecting the natural mode of infection. We selected stages ranging from appressorium-mediated penetration (2.5 hai) to the first occurrence of dead cells, indicative of the switch to necrotrophy (30 hai), as previously described [23]. There were two biological replicates. Each replicate corresponded to RNA extracts from pooled independent biological samples for each condition, to ensure the robustness of our results.

Only hybridization data relevant to *P. parasitica* or sequences of unknown origin were retained for analysis. In total, 4194 sequences (89% of the 4700 probes corresponding to *P. parasitica* sequences or sequences of unknown origin) gave a hybridization signal stronger than the background in at least one condition and were considered to correspond to expressed genes. The oligo-array contained 314 of the 320 putative *P. parasitica-*specific sequences. Significant expression in at least one biological sample was detected for 266 of these sequences, confirming that they corresponded to *P. parasitica*-specific expressed genes. Interestingly, 2864 sequences (61%) and 2987 sequences (64%) were expressed in purified appressoria and during the appressorium-mediated penetration of *A. thaliana* roots (2.5 hai), respectively. Thus, with the inoculation method used for this analysis, a high proportion of transcripts can be detected even at the earliest stages of the interaction.

The Anaïs statistical framework was used to identify differentially expressed sequences [33]. Using a *P*-value threshold of 0.05, 3783 sequences were considered to display differential patterns of accumulation between at least two sets of conditions. Maximum fold-change, defined as the ratio of the maximum and minimum hybridization signal values, was calculated for each sequence. In total, 3471 sequences had a maximum fold-change of more than 2 and 1806 sequences had a maximum fold-change of at least 4.

### Cluster analysis of gene expression patterns

**Figure 1:**
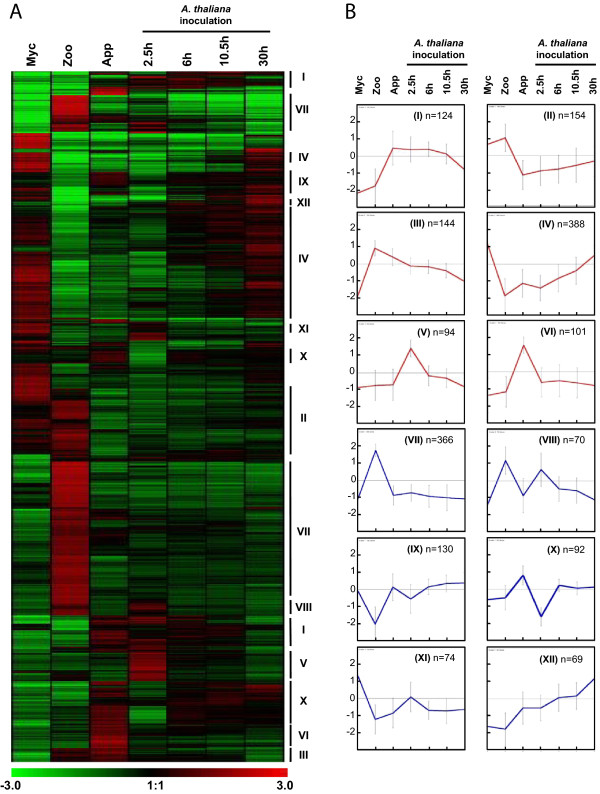
**Hierarchical and K-mean clustering of four-fold expressed genes. (A)** Hierarchical clustering of the 1806 genes modulated by 4-fold. Pearson correlation method with average linkage on conditions was used. Gene expression level is indicated as a Log2 transformation of relative value calculated on gene average signal value. Green and red were used for down-regulated and up-regulated conditions respectively. Lines mark major K-mean groups. **(B)** Expression profiles were K-mean clustered based on euclidean distance. The twelve main K-mean clusters are represented. The center line represents average expression with standard deviation. Number of genes within each cluster is indicated. The six clusters used for subsequent characterization of the molecular events occurring during early infection are colored in red. Myc, mycelium; Zoo, zoospores, App, Appressorium; 2.5 h, *Arabidopsis thaliana* infected roots recovered 2.5 hours after inoculation (appressorium-mediated penetration); 6 h, *A. thaliana* infected roots recovered 6 hours after inoculation (biotrophic growth, two to three cells invaded); 10.5 h, *A. thaliana* infected roots recovered 10.5 hours after inoculation (invasive growth along the stele); 30 h, *A. thaliana* infected roots recovered 30 hours after inoculation (switch to necrotrophy).

The expression patterns of the 1806 sequences displaying at least a four-fold modulation of expression were analyzed. We performed a hierarchical clustering of the expression patterns (Figure 1A) and genes with coordinated expression were grouped into 12 major clusters (K-mean clustering, Figure 1B, Additional file 4: Table S3).

Clusters I (124 sequences), II (154 sequences), III (144 sequences), IV (388 sequences), V (94 sequences), VI (101 sequences) and XX (92 sequences) contained sequences for which expression was modulated during the onset of infection (Figure 1B). Clusters I, II, V, VI and XX contained sequences with a specific modulation of expression (induced or repressed) during early contact with plant cells, as they were detected in purified appressoria and during the first few hours of *A. thaliana* infection (2.5, 6 and 10.5 hai). By contrast, clusters III and IV contained sequences whose accumulation or repression begins in motile zoospores, corresponding to the pre-infection stage.

Sequences from cluster VII (366 sequences) were specifically modulated in zoospores. Interestingly, clusters VIII (70 sequences) and IX (130 sequences) contained sequences with similar patterns of expression in zoospores and in *A. thaliana* roots collected 2.5 hours after inoculation (Figure 1B). This may be due to the contamination of *A. thaliana* root tissues by zoospores, either motile or initiating cyst formation, in the first sample collected for the interaction. This finding is consistent with previous reports indicating that plant infection by motile *P. parasitica* zoospores is an asynchronous process. It thus highlights the advantages of the simplified system previously used to obtain purified appressoria [8].

Cluster XI (74 sequences) grouped together sequences that accumulated preferentially in mycelium (K-mean clustering, Figure 1B). However, most of the sequences found in this cluster also accumulated 2.5 hours after *A. thaliana* inoculation. This may be due to cysts failing to penetrate and the plant and instead initiating mycelial growth around *A. thaliana* roots, as reported during the initial steps of tomato root infection by *P. parasitica* [30].Finally, transcripts from cluster XII (69 sequences) accumulated throughout the development of plant infection (Figure 1B). This accumulation increased during infection, reaching a maximum at the last stage studied (30 hai).

### Validation of the transcriptome data by RT-qPCR

**Figure 2:**
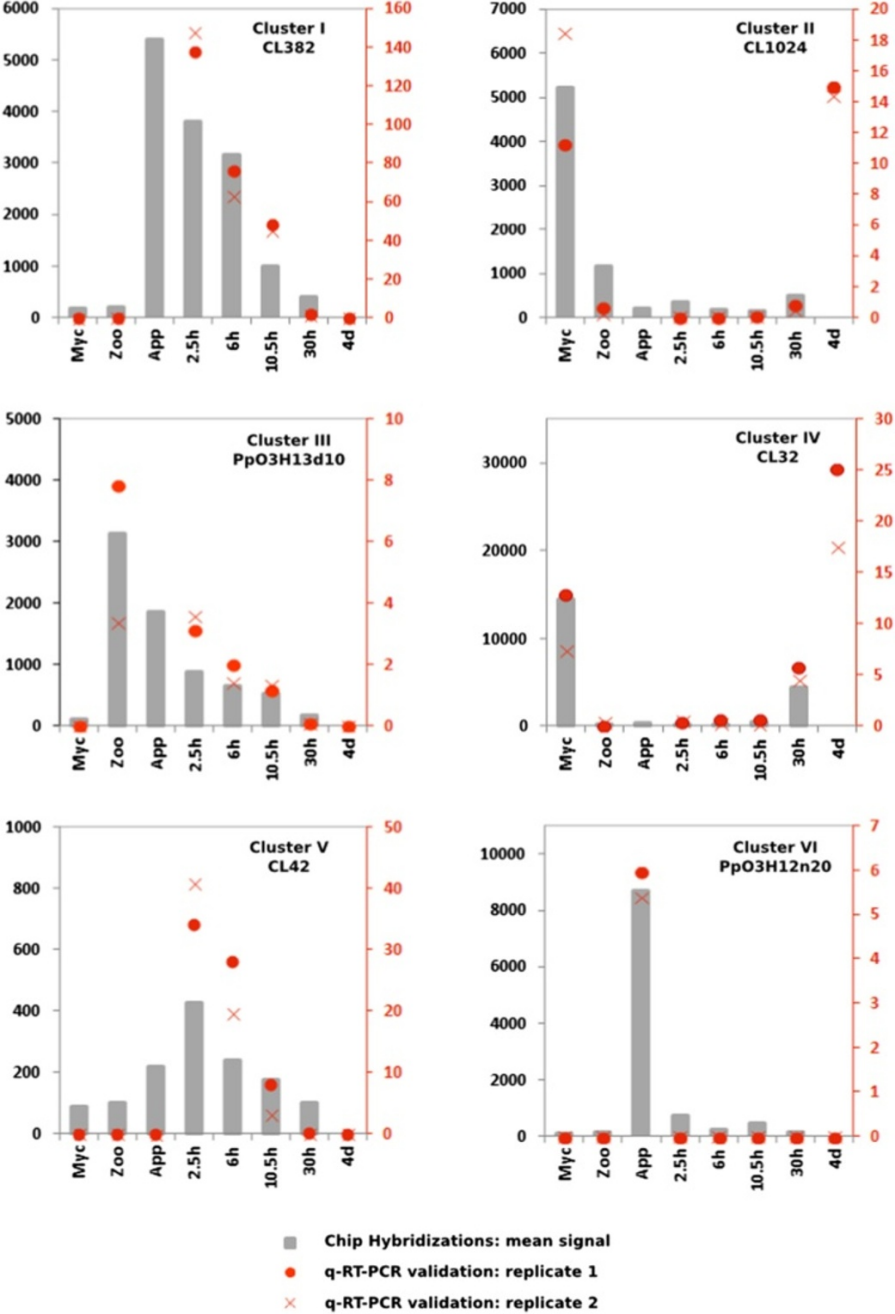
**Quantification of mRNA corresponding to on gene of each of the clusters I to VI.** Relative mRNA levels were quantified by quantitative RT-PCR in biological replicates of samples used in Figure 1. A condition corresponding to *A. thaliana* roots 4 days after inoculation (4d), corresponding to necrotrophic stage of the interaction was added. Data are presented as expression ratios relative to *UBC, WS21* and Mago Nashi reference genes (2^−ΔCT^). Grey bars represent mean signal obtained following oligoarray hybridizations (black left axis). Red point and crosses represent relative expression values obtained for two independent biological replicates analyzed using qRT-PCR (red right axis).

The expression patterns observed on hybridization of the *P. parasitica* oligo array were validated by quantitative RT-PCR. We selected 58 sequences from the six clusters containing sequences with expression modulated during the appressorium-mediated penetration of the host (clusters I, II, III, IV, V and VI; red on Figure 1). A biological sample corresponding to the *A. thaliana*/ *P. parasitica* interaction was added, to monitor expression of the selected sequences during the necrotrophic stage. This sample corresponded to inoculated *A. thaliana* root tissues collected four days after inoculation with zoospores. Forty-four of the 58 genes assessed (75%) displayed a modulation of expression during the onset of the interaction, consistent with the results of array hybridization (Figure 2 and Additional file 5: Table S4). Nine of the 14 sequences for which expression profiling results were not validated by RT-qPCR were from cluster V (Additional file 5: Table S4).

This cluster probably included sequences with artifactual hybridization signals. After we had discarded data for the members of this cluster, the expression patterns for 43 of 48 (90%) sequences were validated by RT-qPCR, demonstrating the robustness of our transcriptome data.

We also used the RT-PCR results to improve our clustering analysis. The grouping of sequences in cluster I, which contained transcripts transiently accumulating during the onset of infection was validated, because eight of the nine sequences had expression patterns consistent with that deduced from oligoarray hybridization (Figure 2, Additional file 5: Table S4). The transcripts of these genes accumulated in smaller amounts during the necrotrophic phase of the *P. parasitica*/*A. thaliana* interaction (4 days after inoculation). This cluster included sequences that accumulated during early infection of the host. The content of clusters III and IV was also validated with all and 8 of the 10 sequences, respectively, displaying the predicted expression pattern (Figure 2, Additional file 5: Table S4). The use of the sample corresponding to necrotrophy also confirmed that the sequences grouped in these clusters accumulated or were repressed transiently in zoospores and during the penetration process. Sequences from cluster II had lower accumulation during early infection than during vegetative mycelial growth and the necrotrophic phase (4 days after inoculation). Nevertheless, seven of these nine sequences also accumulated to low levels in zoospores (Figure 2, Additional file 5: Table S4). This suggests that cluster II could be grouped with cluster IV for a single analysis. Cluster VI contained sequences specifically induced during the penetration of onion epidermis (purified appressoria, Figure 1). This cluster was poorly validated because five of the 10 sequences accumulated during the penetration of both *A. thaliana* roots and onion (Additional file 5: Table S4). This confirms that the results obtained with our simplified inoculation protocol reflect the situation occurring in natural conditions of plant penetration [8]. On the basis of this RT-qPCR analysis, clusters I and VI were grouped together for subsequent analyses.

### Functions of the sequences accumulating during appressorium-mediated plant penetration

**Figure 3:**
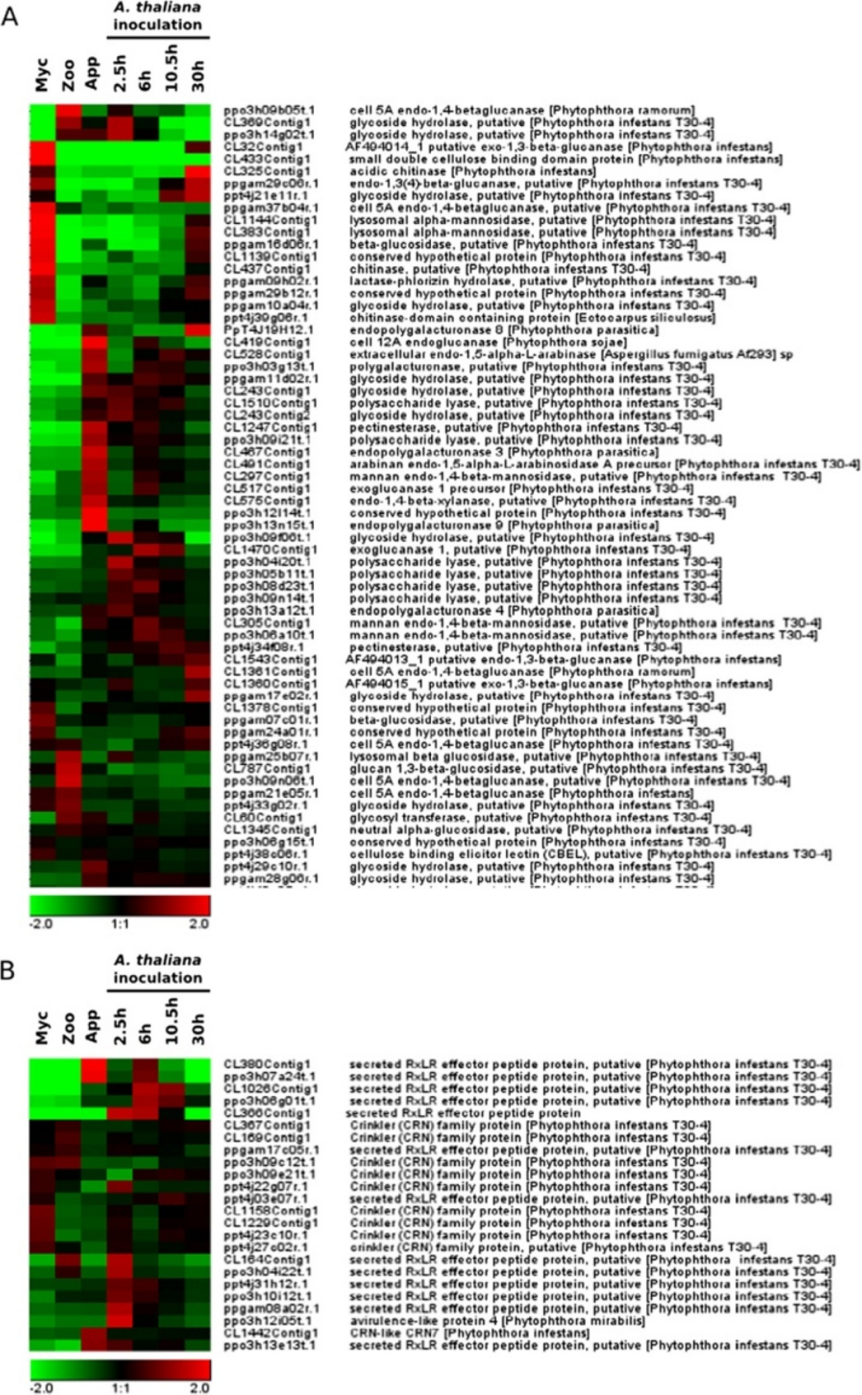
**Hierarchical clustering of the genes encoding cell wall degrading enzymes and cytoplasmic effectors.** Based on GO annotations, sequences corresponding to cell wall degrading enzymes **(A)** and sequences corresponding to RXLR and Crinkler effectors **(B)** are presented. Pearson correlation method with average linkage on conditions was used. Gene expression level is indicated as a Log2 transformation of relative value calculated on gene average signal value. Green and red were used for down-regulated and up-regulated conditions respectively. Myc, mycelium; Zoo, zoospores, App, Appressorium; 2.5 h, *A. thaliana* infected roots recovered 2.5 hours after inoculation; 6 h, *A. thaliana* infected roots recovered 6 hours after inoculation; 10.5 h, *A. thaliana* infected roots recovered 10.5 hours after inoculation; 30 h, *A. thaliana* infected roots recovered 30 hours after inoculation.

We focused our analysis on transcripts modulated during the onset of a compatible interaction (clusters I-VI). A functional annotation of the 20 genes displaying the highest fold-change in each cluster is presented in Table 1. We performed a GO enrichment analysis of the clusters, for annotation purposes. Sequences specifically accumulating during the appressorium-mediated penetration of the host plant (purified appressoria, *A. thaliana* 2.5 hai, 6 hai and 10.5 hai, clusters I and VI) were highly enriched in cell wall-degrading enzymes (CWDEs, Additional file 6: Table S5_I and Additional file 7: Table S5_VI). There were 64 sequences encoding CWDE on the array: 14 and 8 had expression patterns characteristic of clusters I (grouping 124 sequences) and VI (grouping 101 sequences), respectively (Additional file 4: Table S3). Figure 3A shows the expression patterns of all the sequences associated with GO terms relating to cell wall degradation. Thirty CWDEs were found to be preferentially expressed during the first few hours of infection. The other 34 enzymes were expressed in zoospores, mycelium or both mycelium and at the necrotrophic stage of interaction with *A. thaliana*. GO terms associated with the modulation of plant defenses were also overrepresented during the early stages of infection (Additional file 6: Table S5_I). Hence, six of the 14 sequences encoding RXLR effectors present on the oligoarray were present in cluster I. One of these six sequences corresponded to the PSE1 effector, which has recently been demonstrated to contribute to *P. parasitica* pathogenicity [38]. The expression patterns of sequences encoding cytoplasmic (RXLR and CRN) effectors are presented in Figure 3B. Twelve of the RXLR effectors are induced during the penetration process, whereas only a few CRN effector transcripts accumulated at this stage. Other functions relevant to pathogenicity or the elicitation of plant defense responses were also identified in clusters I (2 elicitin-like, 2 NPP1-like and 1 M81 elicitor-like) and VI (a putative protease inhibitor) (Table 1 and Additional file 4: Table S3). GO annotations relating to ribosomes were also enriched in cluster VI, providing evidence for the activation of the translation machinery during early infection (Additional file 7: Table S5_VI).

**Table 1:** **Functional annotation of the sequences from clusters I-VI**

Normalized hydridization signal										Blastx best hit		
CL	Sequence	Myc	Zoo	App	2,5h	6h	10,5h	30h	FC	ID	Description	E value
**I**	CL1533Contig1	102	749	95	10813	1022	712	139	**114**		no hit	
**I**	ppgam36a11r.1	127	131	336	1972	3482	13284	713	**105**	XP_002895059.1	elicitin-like protein INF4 [P. infestans]	3E-10
**I**	ppo3h07a24t.1	97	104	9105	1711	4473	1226	450	**94**	XP_002998010.1	secreted RxLR effector peptide protein, [P. infestans]	2E-13
**I**	CL1408Contig1	120	178	10915	1767	3707	5999	7470	**91**	XP_002896645.1	Amino Acid/Auxin Permease (AAAP) Family [P. infestans]	1E-134
**I**	ppo3h11p12t.1	296	334	4984	26369	10531	11172	2378	**89**		no hit	
**I**	CL262Contig1	196	391	5444	14553	9894	3628	1081	**74**		no hit	
**I**	CL380Contig1	108	120	7702	1192	3335	848	336	**72**	XP_002998010.1	secreted RxLR effector peptide protein, [P. infestans]	5E-19
**I**	CL1335Contig1	333	1011	13535	15140	18621	22234	10825	**67**		no hit	
**I**	ppgam18a12r.1	112	116	119	6434	1077	814	167	**57**		no hit	
**I**	ppo3h08i21t.1	122	128	6892	5236	3381	818	288	**57**	XP_002901148.1	carbohydrate-binding protein, [P. infestans]	2E-13
**I**	ppgam09e02r.1	100	110	116	5457	539	400	126	**55**	XP_001339148.2	PREDICTED: polymerase polyprotein-like [Danio rerio]	3E-53
**I**	CL42Contig1	132	134	7202	5692	3174	681	233	**55**	XP_002901145.1	hypothetical protein PITG_11596 [P. infestans]	1E-09
**I**	ppt4j31g10r.1	126	120	135	5163	639	381	163	**43**		no hit	
**I**	ppo3h12o13t.1	118	154	4926	1188	4607	2779	1464	**42**	XP_002900991.1	conserved hypothetical protein [P. infestans]	2E-32
**I**	ppo3h06d21t.1	190	699	990	3799	7709	4416	2482	**41**	XP_002904652.1	ATP-binding Cassette (ABC) superfamily [P. infestans]	2E-17
**I**	ppo3h05p06t.1	96	96	2003	1513	3273	884	212	**34**	Q56TU4.1	S-adenosylmethionine synthase 1; Daucus carota	1E-13
**I**	CL382Contig1	180	190	5393	3807	3160	987	403	**30**	XP_002901148.1	carbohydrate-binding protein, [P. infestans]	6E-11
**I**	CL1026Contig1	125	144	900	1436	3606	2879	845	**29**	XP_002901054.1	secreted RxLR effector peptide protein, [P. infestans]	2E-15
**I**	CL366Contig1	122	228	274	3032	2717	1029	146	**25**		no hit	
**I**	CL1109Contig1	135	138	3025	1229	3268	2982	2582	**24**	XP_002898673.1	ribonuclease, [P. infestans]	1E-89
**II**	CL1024Contig1	5212	1169	195	341	160	154	481	**34**	XP_500020.1	YALI0A12705p [Yarrowia lipolytica] emb	1E-13
**II**	ppgam23f03r.1	3620	1975	358	224	136	126	158	**29**	XP_002895853.1	sporangia induced phosphatidyl inositol kinase [P. infestans]	1E-142
**II**	CL1233Contig1	3966	6902	240	430	353	738	1651	**29**	XP_002899328.1	Ca2+−transporting ATPase endoplasmic reticulum type, [P. infestans]	0
**II**	CL1149Contig1	903	2978	308	207	297	436	462	**14**	XP_002905211.1	protein kinase, [P. infestans]	0
**II**	CD051670.1.1	1313	2611	2387	248	185	207	625	**14**		no hit	
**II**	CL1407Contig1	10595	4542	757	1385	2211	3020	2338	**14**	XP_002908946.1	dihydroflavonol-4-reductase, [P. infestans]	1E-159
**II**	ppt4j11b07r.1	636	2408	175	259	352	341	343	**14**	XP_002903886.1	conserved hypothetical protein [P. infestans]	3E-99
**II**	CL772Contig1	4458	2341	605	336	991	969	1198	**13**	XP_002997937.1	conserved hypothetical protein [P. infestans]	0
**II**	CL864Contig1	9998	4700	787	1220	1070	1225	2643	**13**	XP_002898044.1	conserved hypothetical protein [P. infestans]	0
**II**	CL112Contig2	5791	4284	1116	2745	2166	1535	463	**13**	XP_002895909.1	ATP-binding Cassette (ABC) Superfamily [P. infestans]	0
**II**	ppgam04e11r.1	649	2816	231	262	328	349	557	**12**		no hit	
**II**	CL1478Contig1	771	2708	235	250	347	341	514	**12**	XP_002907456.1	conserved hypothetical protein [P. infestans]	7E-76
**II**	ppgam07d02r.1	634	2263	196	293	225	275	345	**12**	XP_002998017.1	transmembrane protein, [P. infestans]	7E-71
**II**	CL471Contig1	5487	4101	582	848	477	756	1567	**12**	XP_002896830.1	conserved hypothetical protein [P. infestans]	1E-100
**II**	CL1485Contig1	1608	838	158	143	186	180	347	**11**	XP_002897750.1	short/branched chain specific acyl-CoA dehydrogenase, [P. infestans]	1E-125
**II**	CL349Contig1	1340	4691	549	502	543	431	729	**11**	XP_002909307.1	conserved hypothetical protein [P. infestans]	0
**II**	ppgam33a03r.1	2179	1553	202	508	579	938	701	**11**	XP_002905400.1	L-aminoadipate-semialdehyde dehydrogenase, [P. infestans]	1E-128
**II**	CL874Contig1	1039	2304	216	452	351	573	601	**11**	XP_002997338.1	HECT E3 ubiquitin ligase, [P. infestans]	7E-89
**II**	CL176Contig1	2239	5389	710	506	573	549	834	**11**	XP_002897342.1	conserved hypothetical protein [P. infestans]	8E-61
**II**	ppt4j38b11r.1	681	2149	332	246	230	202	432	**11**	XP_002896848.1	conserved hypothetical protein [P. infestans]	1E-108
**III**	ppo3h14g02t.1	142	11120	10795	18486	7479	2452	503	**130**	XP_002997786.1	glycoside hydrolase, [P. infestans]	1E-43
**III**	ppo3h14f01t.1	126	5446	7895	14463	5042	1485	437	**115**		no hit	
**III**	CD051442.1.1	218	15744	14847	23012	17903	9763	5659	**105**		no hit	
**III**	CL369Contig1	122	11151	4457	12379	4521	1277	268	**102**	XP_002997786.1	glycoside hydrolase, [P. infestans]	1E-138
**III**	CD051500.1.1	157	15890	5422	7601	5856	5131	1149	**101**		no hit	
**III**	CL306Contig1	148	14591	3542	5108	3343	3089	710	**99**	XP_002898718.1	conserved hypothetical protein [P. infestans]	1E-146
**III**	CL804Contig1	130	9313	6270	2748	2823	2240	1429	**72**	XP_002904112.1	sulfatase-like protein [P. infestans]	1E-132
**III**	CL515Contig1	131	7782	2639	1063	1153	1103	519	**59**	XP_002904421.1	conserved hypothetical protein [P. infestans]	0
**III**	CD051686.1.1	135	6845	2453	1660	868	844	290	**51**		no hit	
**III**	CL1187Contig1	181	9106	1049	3912	5197	6878	5043	**50**	XP_002896478.1	poly [ADP-ribose] polymerase, [P. infestans]	1E-174
**III**	CF891673.1.1	157	5465	5871	2105	4405	2699	1995	**37**		no hit	
**III**	CL232Contig1	200	7317	3970	3044	4343	2281	3612	**37**	XP_002904949.1	conserved hypothetical protein [P. infestans]	1E-168
**III**	CL1195Contig1	135	4941	2491	3037	2412	1683	724	**37**	XP_002895053.1	conserved hypothetical protein [P. infestans]	1E-129
**III**	CL181Contig1	276	7495	9615	1909	1423	1212	1047	**35**	XP_002900508.1	conserved hypothetical protein [P. infestans]	1E-119
**III**	ppo3h09b05t.1	172	5371	703	1742	819	937	463	**31**	ABG80552.1	cell 5A endo-1,4-betaglucanase [P. ramorum]	2E-97
**III**	ppo3h07h24t.1	112	3238	1398	543	840	680	469	**29**	XP_002906516.1	transmembrane protein, [P. infestans]	1E-117
**III**	ppo3h13d10t.1	115	3128	1867	885	647	518	171	**27**	XP_002998505.1	GPI-anchored serine-rich elicitin INL3b-like protein [P. infestans]	1E-38
**III**	CL449Contig1	125	3223	2186	1589	1510	843	410	**26**	XP_002898095.1	conserved hypothetical protein [P. infestans]	7E-96
**III**	ppt4j29b11r.1	158	3765	2184	1048	557	849	399	**24**	XP_002902649.1	Drug/Metabolite Transporter (DMT) Superfamily [P. infestans]	1E-108
**III**	CL330Contig1	1867	23600	43890	40265	40618	35214	14295	**24**	XP_002904340.1	conserved hypothetical protein [P. infestans]	1E-102
**IV**	CL8Contig1	44525	163	325	452	825	9662	7144	**274**	XP_002899844.1	conserved hypothetical protein [P. infestans]	1E-152
**IV**	CL639Contig1	22961	606	806	221	192	166	413	**139**	ABG23233.1	unknown [Hyaloperonospora parasitica]	1E-36
**IV**	ppo3h02e04t.1	19582	157	3139	1151	2639	7487	7743	**124**	XP_002900352.1	Proton-dependent Oligopeptide Transporter (POT) Family [P. infestans]	8E-98
**IV**	ppgam01h01r.1	17201	173	339	204	164	142	881	**121**	XP_002901432.1	conserved hypothetical protein [P. infestans]	6E-33
**IV**	ppo3h10m18t.1	25224	224	6090	1667	2610	4991	19398	**113**		no hit	
**IV**	CL90Contig1	13934	167	1552	489	7712	8560	18029	**108**	XP_002999240.1	pyrophosphate vacuolar membrane proton pump, [P. infestans]	0
**IV**	CL32Contig1	14467	189	275	148	258	429	4440	**97**	AAM18483.1	AF494014_1 exo-1,3-beta-glucanase [P. infestans]	0
**IV**	ppgam24d04r.1	13873	149	200	667	295	697	973	**93**	XP_002907089.1	protein kinase, [P. infestans]	1E-127
**IV**	CL210Contig1	12224	133	178	647	362	905	11665	**92**	XP_002909387.1	conserved hypothetical protein [P. infestans]	7E-69
**IV**	ppgam36c10r.1	16676	187	4612	1365	566	1463	8770	**89**	XP_002904661.1	Major Facilitator Superfamily (MFS) [P. infestans]	1E-126
**IV**	CL119Contig1	10489	126	163	144	246	461	494	**84**	XP_002900778.1	Annexin (Annexin) Family [P. infestans]	1E-166
**IV**	ppgam37e05r.1	15664	342	205	390	190	188	2589	**83**	XP_002907653.1	oxidoreductase, [P. infestans]	4E-85
**IV**	CL71Contig1	10898	137	1568	339	1504	4481	9186	**79**	ABH11757.1	elicitin-like protein 6 precursor [P. nicotianae]	4E-44
**IV**	ppo3h07a23t.1	9263	118	124	161	162	175	1752	**78**	XP_002907297.1	P-type ATPase (P-ATPase) Superfamily [P. infestans]	1E-120
**IV**	CL92Contig1	12589	213	871	357	5799	12282	16142	**76**	XP_002998388.1	carbohydrate-binding protein, [P. infestans]	1E-127
**IV**	ppgam07a10r.1	8342	249	671	275	1663	9237	18817	**76**	XP_002903354.1	mucin-like protein [P. infestans]	8E-38
**IV**	ppgam02h08r.1	4632	136	625	454	3462	10250	8890	**75**	XP_002896569.1	glucosylceramidase, [P. infestans]	1E-109
**IV**	ppt4j09a01r.1	8222	115	199	259	220	399	8676	**75**	XP_002909387.1	conserved hypothetical protein [P. infestans]	2E-46
**IV**	ppgam18e03r.1	15537	715	353	538	227	207	2704	**75**	XP_002902980.1	acyl-CoA synthetase short-chain family member, [P. infestans]	1E-124
**IV**	CL758Contig1	12392	169	311	676	908	2489	3671	**73**	XP_002904197.1	glucosylceramidase, [P. infestans]	1E-156
**V**	ppo3h06f02t.1	409	777	214	8582	1544	1290	509	**40**	XP_002997773.1	conserved hypothetical protein [P. infestans]	7E-78
**V**	ppgam12h06r.1	781	868	171	5385	359	274	138	**39**		no hit	
**V**	ppgam31f01r.1	119	197	159	2339	520	353	164	**20**		no hit	
**V**	ppt4j25b05r.1	97	176	126	1823	424	369	127	**19**	AAR21576.1	heat shock protein 70 [P. nicotianae]	2E-20
**V**	CL1505Contig1	480	596	349	5757	3617	1585	1274	**17**	XP_002895485.1	ATP-binding Cassette (ABC) superfamily [P. infestans]	1E-136
**V**	ppt4j21a06r.1	445	118	199	1866	303	357	311	**16**	XP_002895988.1	maltose O-acetyltransferase, [P. infestans]	5E-86
**V**	ppgam36f04r.1	108	116	171	1707	275	240	140	**16**		no hit	
**V**	ppgam17d07r.1	159	458	362	2083	608	514	333	**13**	XP_002906389.1	conserved hypothetical protein [P. infestans]	2E-65
**V**	CL344Contig1	138	179	483	1684	534	446	257	**12**		no hit	
**V**	ppgam04f05r.1	128	167	462	1554	521	297	166	**12**		no hit	
**V**	ppo3h02b24t.1	112	407	356	1344	495	796	671	**12**	XP_002895175.1	conserved hypothetical protein [P. infestans]	5E-51
**V**	ppo3h06n03t.1	129	135	526	1544	284	204	175	**12**	NP_001063268.1	Os09g0438100 [Oryza sativa Japonica Group] dbj	0,000009
**V**	ppt4j01f03r.1	106	122	165	1257	236	187	145	**12**	XP_002903318.1	conserved hypothetical protein [P. infestans]	1E-15
**V**	ppo3h03i13t.1	161	608	253	1692	729	454	216	**11**	XP_002904470.1	conserved hypothetical protein [P. infestans]	5E-97
**V**	ppo3h01k13t.1	126	418	348	1275	506	395	230	**10**	XP_002903425.1	conserved hypothetical protein [P. infestans]	3E-45
**V**	ppt4j36c04r.1	291	127	168	1285	233	258	241	**10**	XP_002182839.1	predicted protein [Phaeodactylum tricornutum CCAP 1055/1]	4E-51
**V**	ppo3h12p16t.1	564	1450	1037	5589	1138	1127	581	**10**	XP_002898514.1	conserved hypothetical protein [P. infestans]	1E-36
**V**	ppo3h03o24t.1	240	119	230	1158	360	368	185	**10**		no hit	
**V**	ppt4j38e02r.1	102	102	100	943	200	196	117	**9**		no hit	
**V**	ppgam22f11r.1	122	120	153	1082	249	223	145	**9**	XP_002896344.1	conserved hypothetical protein [P. infestans]	2E-14
**VI**	ppo3h12n08t.1	104	106	37783	103	124	120	116	**365**		no hit	
**VI**	ppo3h10g01t.1	113	131	9703	121	207	111	112	**87**		no hit	
**VI**	ppo3h12n20t.1	104	128	8715	707	225	478	128	**84**	XP_002909505.1	mannitol dehydrogenase, [P. infestans]	5E-41
**VI**	ppo3h13j21t.1	92	94	4826	93	122	112	96	**52**		no hit	
**VI**	CL613Contig1	117	129	5082	224	1021	1359	1803	**43**	XP_002904550.1	folate-Biopterin Transporter (FBT) family [P. infestans]	0
**VI**	ppo3h03f02t.1	132	996	5251	350	169	144	142	**40**	XP_002904736.1	conserved hypothetical protein [P. infestans]	2E-69
**VI**	ppo3h05l14t.1	95	173	3749	261	179	118	114	**39**	XP_002905279.1	cleavage induced hypothetical protein [P. infestans]	5E-86
**VI**	CL419Contig1	139	323	5306	737	1879	924	595	**38**	ABB22029.1	cell 12A endoglucanase [P. sojae]	1E-117
**VI**	ppo3h06d03t.1	112	124	4159	443	816	580	173	**37**	XP_002904437.1	conserved hypothetical protein [P. infestans]	1E-112
**VI**	ppt4j22c09r.1	118	593	3355	2610	422	405	321	**28**	XP_002909523.1	Amino Acid-Polyamine-Organocation (APC) Family [P. infestans]	1E-115
**VI**	ppo3h04p13t.1	113	118	2658	565	195	151	129	**24**	XP_002901038.1	carbohydrate-binding protein, [P. infestans]	1E-58
**VI**	CL118Contig1	105	106	2438	176	140	160	118	**23**	XP_002902444.1	conserved hypothetical protein [P. infestans]	1E-107
**VI**	CL999Contig1	117	131	2593	402	881	527	270	**22**	XP_002901373.1	conserved hypothetical protein [P. infestans]	1E-86
**VI**	CL1422Contig1	138	172	3038	797	222	254	913	**22**	XP_002904844.1	ATP-binding Cassette (ABC) Superfamily [P. infestans]	1E-165
**VI**	CL317Contig1	160	124	2693	276	585	516	953	**22**	XP_002901401.1	conserved hypothetical protein [P. infestans]	7E-26
**VI**	CL1406Contig1	105	112	1979	156	169	164	117	**19**	ABG23232.1	N-acetyltransferase-like protein [Hyaloperonospora parasitica]	3E-49
**VI**	CL448Contig1	237	228	4124	1663	1472	745	545	**18**	XP_002900313.1	cutinase, [P. infestans]	1E-103
**VI**	CL1513Contig1	124	124	2132	264	650	571	264	**17**	XP_002903927.1	pectin lyase, [P. infestans]	2E-61
**VI**	CL1176Contig1	106	116	1784	152	122	124	108	**17**	XP_002999045.1	conserved hypothetical protein [P. infestans]	1E-142
**VI**	ppo3h13a15t.1	97	100	1585	101	119	110	102	**16**	XP_002518625.1	conserved hypothetical protein [Ricinus communis]	1E-51

Annotation proposed for the 20 genes with the highest fold change in each cluster (CL) is presented. For all sequences, normalized hybridization signal in each condition (mean of the two biological replicates) is indicated. Maximum fold change (max FC) observed between the different biological samples and BlastX best hit result (E value < 1E-05) against NCBI non-redundant (NR) protein database are presented.

An analysis of clusters I and VI showed these clusters to contain sequences involved in protein and amino-acid metabolism, including four proteases and four putative amino-acid transporters. Similarly, eight and three sequences encoding functions involved in the detoxification of plant toxic metabolites (ABC transporters and major facilitator superfamily members) were identified in clusters I and VI, respectively (Additional file 4: Table S3). Interestingly, sequences relating to sugar metabolism were poorly represented in these clusters. Cluster I included a sequence corresponding to mannose 6P isomerase, a pyruvate kinase and a NAD-dependent malic enzyme. A single sequence encoding a malate dehydrogenase was identified in cluster VI (Additional file 4: Table S3). A similar situation was observed for sequences relating to fatty-acid catabolism. Only one sequence encoding a long-chain fatty acid CoA ligase and one sequence encoding an acyl-CoA dehydrogenase were identified in clusters I and VI, respectively (Table 1 and Additional file 4: Table S3).

The prominent functions identified for sequences from cluster III (sequences accumulating in zoospores and during host penetration, 144 sequences) were mostly similar to those identified in clusters I and VI. GO enrichment analysis highlighted the accumulation of sequences with functions relating to ribosome biogenesis (Additional file 8: Table S5_III). However, a detailed analysis of this cluster revealed the occurrence of three additional cell wall-degrading enzymes and numerous infection-associated sequences, including five M81-like sequences, two OPEL-like sequences, one EPIC-like sequence and one elicitin (Table 1 and Additional file 4: Table S3). Three sequences encoding functions involved in the evasion of toxic molecules (ABC transporters and major facilitator superfamily members) were observed. Finally, this cluster also contained four proteases and one amino acid/auxin permease, confirming the importance of amino-acid uptake during early infection. Only two glycolytic enzymes (glucokinase and a glyceraldehyde 3P dehydrogenase) and a fatty-acid metabolism-related short-chain dehydrogenase were identified in this cluster.

Cluster V, which contained 94 sequences specifically expressed during the penetration of *A. thaliana* roots, but not onion epidermis, was not supported by RT-qPCR experiments, suggesting that this cluster should be considered with caution. Moreover, most of the sequences in this cluster lacked robust annotation. Nevertheless, like clusters I and VI, this group contained various pathogenicity-related sequences, including a range of apoplastic and cytoplasmic effectors: four RXLR effectors, two CRNs, one elicitin-like sequence, one IPI-like sequence, one NPP1-like sequence and one protease (Table 1, Additional file 9: Table S5_V and Additional file 4: Table S3).

### Functions of sequences repressed during appressorium mediated penetration of the host

An analysis of the sequences of cluster II (154 sequences specifically repressed during appressorium-mediated penetration) revealed enrichment for functions involved in signaling (Additional file 10: Table S5_B). A more detailed analysis identified 15 potential protein kinases, including three phosphatidyl-inositol kinases and two MAP-kinases (Table 1 and Additional file 4: Table S3). Sequences relating to lipid metabolism (2 acyl-CoA dehydrogenases, 2 acyl-CoA ligases) and ubiquitin-mediated protein degradation (2 E3 ubiquitin ligases and one ubiquitin-like protein) were also identified in this cluster.

For sequences repressed both in zoospores and during the penetration process (cluster IV, 388 sequences), most of the associated GO terms identified related to metabolism (Additional file 11: Table S5_IV). Primary metabolism was globally downregulated, as indicated by the numbers of sequences relating to glycolysis/neoglucogenesis, the pentose phosphate pathway, lipid metabolism, and their overall repression level (Table 1 and Additional file 4: Table S3). Similarly, the ubiquitin-proteasome pathway (12 proteasome components and 9 sequences associated with the proteolytic process) was repressed during early infection (Table 1 and Additional file 4: Table S3).

Surprisingly, an analysis of cluster IV led to the identification of functions otherwise overrepresented during the penetration process. The sequences of this cluster included sequences encoding enzymes involved in cell wall degradation (15 sequences), proteases (13 sequences), elicitin-like proteins (4 sequences) and potential ABC transporters (8 sequences, Table 1 and Additional file 4: Table S3). These findings suggest that multiple functions are achieved by specific proteins during the penetration process, with these proteins being downregulated during the other steps of the *P. parasitica* life cycle.

### An RXLR effector is expressed in the appressorium and infectious hyphae

**Figure 4:**
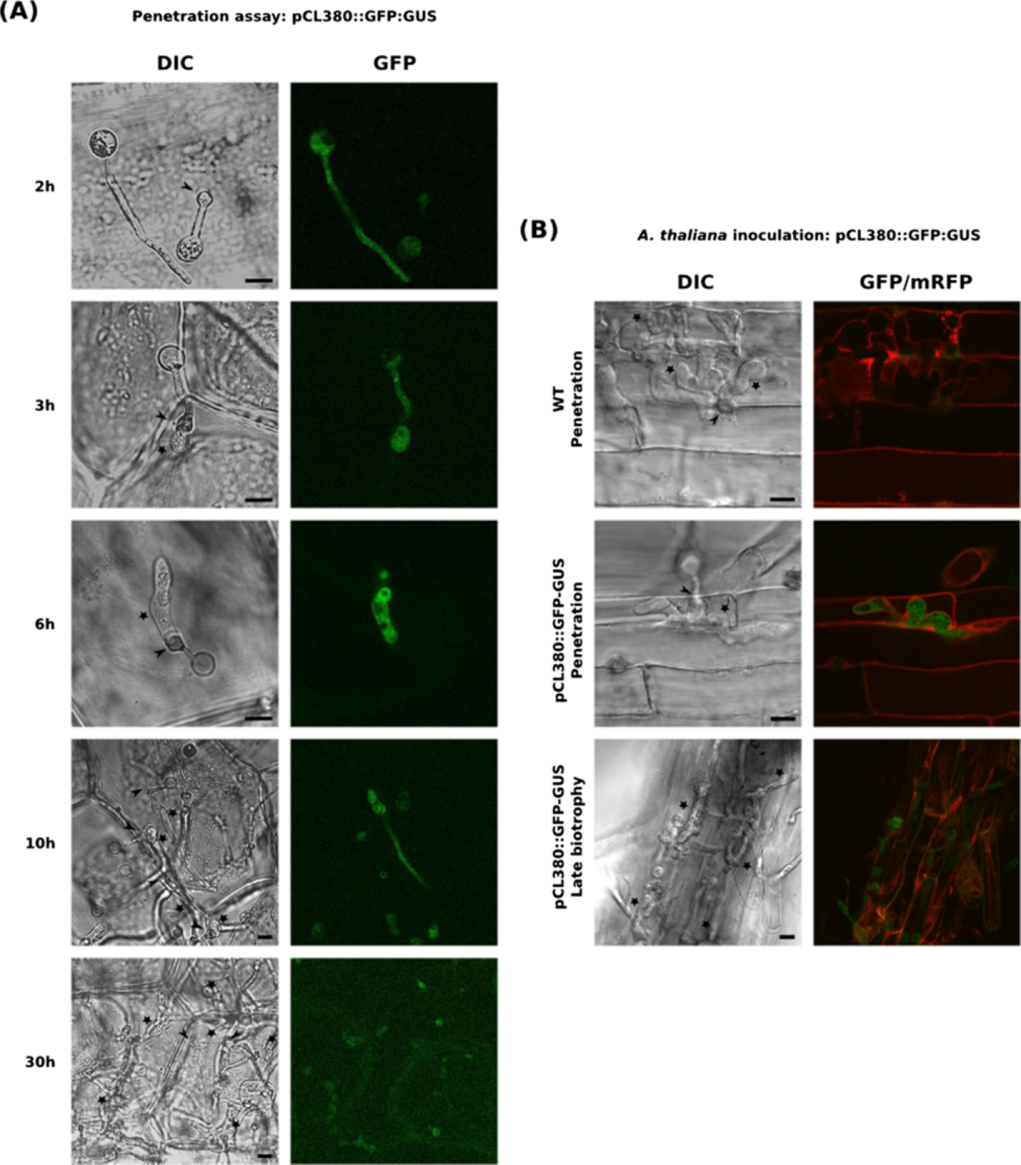
***P. Parasitica*** **appressorium transiently express a RXLR encoding effector during the penetration process. (A** **and** **B)** Expression pattern of the CL380 RXLR encoding gene using a pCL380::GFP-GUS fusion. **(A)** Kinetic of early infection steps using a simplified penetration assay based on onion epidermis. GFP fluorescence from a *P. parasitica* strain carrying the pCL380::GFP-GUS construct was monitored 2 to 30 hours after inoculation of zoospores on onion epidermis. **(B)** Analysis of the CL380 expression pattern during early *A. thaliana* infection. *A. thaliana* plantlets where inoculated with zoospores from a *P. parasitica* strain carrying the pCL380::GFP-GUS construct and fluorescence was monitored during infection of the first cell (6 hours after inoculation for the presented image) and late biotrophic phase (24 hours after inoculation for the proposed image). Plant cell membranes (red) are visualized with the mCherry plasma membrane marker pm-RB. A representative image for each stage is presented. GFP and mCherry fluorescence was visualized using a confocal laser scanning microscope. Bars: 10 μM; Arrows: appressoria; Stars: Infectious hyphae.

A significant number of transcripts encoding RXLR effectors were found to accumulate during host penetration. These proteins are generally thought to be delivered into the plant cytoplasm via haustoria [39]. We analyzed the expression pattern of an RXLR sequence from cluster I. This gene, represented by the unisequence CL380, was chosen for study because it was strongly expressed during the penetration process (Table 1). *P. parasitica* transformants expressing a pCL380::GFP-GUS transcriptional fusion were obtained and used in the onion epidermis-based simplified penetration assay, for a precise analysis of CL380 expression during penetration. Two independent transformants gave similar GFP expression patterns. Expression was detected in germinated cysts (2 hai) and a faint GFP signal was observed in the cyst and germ tube (Figure 4A, 2 h). Fluorescence remained weak in germinated cysts differentiating into appressoria (Figure 4A, 2 h). The GFP signal increased 3 hours after inoculation, at the onset of penetration, and peaked six hours after inoculation (Figure 4A, 3 h and 6 h). The GFP signal was localized in infectious hyphae, and the cysts and appressoria appeared to have no cytoplasm at this stage (Figure 4A, 6 h). The GFP signal decreased 10 hours after inoculation, when the infectious hyphae grew into the cells and became weakly detectable, 30 hours after inoculation, in heavily colonized tissues. As GFP is stable for almost 24 h, the decrease in fluorescence intensity is accounted for by both transcription arrest and the dilution of the existing GFP in the developing infectious hyphae. This result confirmed that the CL380 effector accumulated transiently during the penetration process. Zoospores from transformants expressing the pCL380::GFP-GUS transcriptional fusion were then used to inoculate *A. thaliana* plantlets. A transgenic line accumulating a plasma membrane-targeted mCherry fluorescent protein was used to visualize the boundaries of the plant cell cytoplasm. We observed mCherry fluorescence around the infectious hyphae, suggesting that *P. parasitica* invades plant cells by growing between the plasma membrane and the cell wall (Figure 4B). As in onion epidermis, GFP fluorescence was observed just after penetration, in the infectious hyphae of the transgenic *P. parasitica* strain, whereas no fluorescence was detected with the wild-type strain (8 hours post inoculation for the image presented, Figure 4B). This result confirms that this RXLR-encoding gene is expressed during penetration of the first plant cell. The fluorescence subsequently decreased but continued to be observed in some areas of the infected roots at the late biotrophy stage (24 hours post inoculation for the image presented, Figure 4B). This may be due, in part, to the stability of GFP, which delays the decrease in fluorescence. Moreover, as *P. parasitica* infection is an asynchronous process, the infection events observed 30 hours after the inoculation of *A. thaliana* may correspond to different biological stages. Some parts of the roots may undergo early biotrophic *P. parasitica* colonization, even 30 hours after inoculation. It was more difficult to detect mCherry fluorescence at this stage, which suggests that plant cells are already affected by *P. parasitica* colonization. Taken together, these results confirm that *P. parasitica* transiently expresses RXLR effectors during the penetration process.

## Discussion

We developed a custom oligoarray, using all the *P. parasitica* sequences available at the start of this project. These sequences were obtained in a range of biological conditions, including plant infection. A set of 5451 unisequences was generated by EST clustering and assembly and oligonucleotides were designed for all but 24 of these sequences. Overall, 87% of these sequences were attributed to *P. parasitica* or were of unknown origin. Based on the recently released complete *P. parasitica* genome sequence, we determined that the array made it possible to study the expression of about 20% of the predicted genes of this species. The oligoarray was used to monitor gene expression during the onset of infection. The objective was to characterize the genetic program activated during appressorium differentiation and the penetration of the first host cells. RNA recovered from vegetative cultures, pre-infection structures (motile zoospores and cysts differentiating into appressoria on onion) and samples collected from the plant (corresponding to *A. thaliana* tissues collected from 2.5 to 30 hours after infection) was used to characterize the initial steps of infection. We detected 89% of the sequences in at least one set of conditions. This high rate of detection is slightly higher than the 69%, 79% and 75% reported during analyses of the early infection process in *P. infestans*, *P. sojae* and *P. capsici,* respectively [19–21]. This result is unsurprising, because EST sequences were used for the design of the array, rather than genome-based predicted open reading frames. In particular, 64% of the sequences were detected 2.5 hours after the inoculation of *A. thaliana* roots, which is not surprising because 20% of the ESTs used for the assembly of sequences analyzed with the array were obtained from germinated cysts with appressoria [8]. Thus, our methods were effective for detecting sequences expressed at early stages of infection. A large proportion of the sequences were differentially expressed, with 74% and 38% displaying modulations of at least two-fold and at least four-fold, respectively, between at least two sets of conditions. This proportion is higher than the proportion of genes displaying a two-fold modulation of expression (46%) described by Judelson and coworkers, who analyzed the events from sporangium cleavage to the *in vitro* differentiation of appressoria [19]. This difference may reflect the large proportion of genes with expression modulated during plant penetration, a biological condition not tested in the transcriptome analysis for *P. infestans*. Other studies on fungi have generated findings consistent with this conclusion. Only 29% of *Magnaporthe grisea* genes have been shown to display a two-fold modulation of expression during *in vitro* appressorium differentiation, whereas up to 44% of *Colletotrichum higginsianum* genes were found to display a four-fold modulation of expression in a transcriptome analysis of samples corresponding to plant infection [40, 41].

A clustering analysis identified five highly validated clusters containing sequences modulated during appressorium-mediated penetration of the host. Sequences accumulating transiently during the penetration of the first plant cells, some of which were already accumulating in zoospores before infection, were identified. Similarly, sequences displaying transient downregulation upon host penetration were also identified. Such sequences, displaying specific downregulation in appressoria, were not identified in *P. infestans* germinated cysts with appressoria obtained *in vitro* [19]. The perception of environmental cues at the plant surface may contribute to this downregulation.

An analysis of the predicted function of the genes transiently expressed or repressed provided insight into the genetic program activated during early infection. The cluster grouping together sequences specifically repressed during the first few hours of infection (cluster II) contained a large number of genes relating to lipid and sugar metabolism. Consistent with this observation, only a few sequences relating to sugar or lipid degradation were identified in cluster I, which contained sequences transiently accumulating during the penetration process. By contrast, many sequences relating to protein degradation and amino-acid uptake were identified among the sequences accumulating at this stage. Taken together, these results suggest that *P. parastica* may use amino-acid uptake from the plant as a carbon source, as soon as penetration occurs. By contrast, sugar and lipid metabolism may be repressed at early stages of infection. Jupe and coworkers recently reported an enrichment in functions relating to gene expression and metabolism during the biotrophic phase of tomato infection by *P. capsici* [21]. They suggested that amino-acid uptake from the plant would not be favored by *P. capsici* at early stages of infection. This study provided no information about sugar and lipid metabolism but, as we obtained conflicting results, additional studies are required to determine which carbon sources are used by *Phytophthora* species during early stages of infection.

A large number of sequences relating to signaling were identified in the cluster grouping together sequences transiently repressed during penetration, within which protein kinases were particularly abundant. In addition, few, if any, signaling-related sequences were identified among the cellular functions for sequences accumulating during penetration of the host plant by *P. parasitica*. As calcium is known to be required for appressorium differentiation in *P. infestans*, related signaling pathways should have been detected during penetration [10]. The absence of these functions among the penetration-specific genes may be due to the corresponding transcripts and/or proteins accumulating in zoospores before infection, in avoid the need for *de novo* expression in appressoria and young infectious hyphae. Indeed, the penetration process occurs very rapidly after zoospore germination in *P. parasitica* [8, 23]. Consistent with this hypothesis, several protein kinases, including a calcium-dependent kinase, and transcription factors were observed among the cluster II sequences transiently expressed in zoospores. Thus, the zoospore, in addition to its role in dissemination, may be considered to be a pre-infection stage expressing important functions required for the penetration process.

An analysis of the sequences transiently accumulating during penetration highlighted functions that have been reported to influence the behavior of the interaction. The proteins concerned included a set of proteins triggering the necrosis of plant tissues and including elicitins, Nep-like proteins and proteins homologous to M81 elicitors. Sequences encoding protease inhibitors and transporters putatively involved in the efflux of toxic plant molecules were also observed. Such sequences have been reported to accumulate in *P. infestans* germinated cysts and probably constitute general weapons in the arsenal of *Phytophthora* spp. [18, 19]. Cell wall-degrading enzymes were particularly abundant among the transcripts transiently accumulating during the first few hours of infection. Judelson and coworkers also detected numerous CWDEs in *P. infestan*s germinated cysts [19]. We also noticed that CWDEs displayed very unusual expression patterns. Some enzymes were specific to penetration, whereas others were expressed in the mycelium or during the necrotrophic phase of the interaction. This result confirms our previous hypothesis that a specific set of *P. parasitica* CWDEs may be mobilized to soften the plant cell wall and facilitate penetration [8]. The finding that other CDWE genes are expressed in vegetative cultures or during the necrotrophic phase of the interaction suggests that necrotrophy is related to saprophytic growth and that this second set of enzymes is used to obtain sugars from the walls of dead cells.

Finally, most of the RXLR effectors represented on the array displayed transient expression during penetration. This finding was not unexpected, because most of these sequences originated from appressorium-derived cDNAs [8]. Nevertheless, this result indicates that, as observed for CWDEs, a specific set of effectors is activated during penetration. No such expression pattern was not observed for transcripts encoding CRN proteins, suggesting different roles for these two classes of cytoplasmic effectors. Previous studies reported the detection of transcripts encoding secreted effectors in appressoria from *Phytophthora* species such as *P. infestans*, *P. sojae*, and *P. capsici* and in appressoria from ascomycetes, such as *Magnaporthe grisea* and *Colletotrichum higginsianum* [19, 21, 40, 41]. However, the secretion of *Phytophthora* cytoplasmic effectors has been documented only in haustoria to date. By using a transcriptional fusion with the GFP reporter gene, we were able to detect the expression of an effector in the appressorium and infectious hyphae during the penetration process. Our work thus suggests that appressoria and infectious hyphae are not only involved in plant penetration, they may also act as secretory organs for the transfer of cytoplasmic effectors into the host. These penetration-specific effectors may be involved in manipulating plant cells to facilitate establishment of the pathogen. Wang and coworkers described successive waves of effector expression during plant infection by *P. sojae* [42]. These authors suggested a relay between these successive waves, interfering with plant immunity at various levels. We have shown that the PSE1 effector, which is transiently expressed during penetration, can interfere with auxin physiology to facilitate plant infection [38]. Additional functional analyses of effectors transiently expressed during the onset of infection are required to determine whether this specific set of proteins plays a particular role in modulating plant development.

This study provides new insight into the process by which *Phytophthora* species penetrate their hosts. Based on our results for about one fifth of the gene content of this species, we propose several hypotheses concerning the biology of the infection process of *P. parasitica*. Future projects, making use of the full genome sequence, which is now available, will complete this analysis and should make it possible to test our hypotheses.

## Conclusion

By using precisely calibrated interaction systems, we characterized the genetic program activated by *P. parasitica* during the initial infection of *A. thaliana* cells. Expression of a series of genes is transiently modulated during the penetration of the host. These modulations account for a specific developmental program occurring at the penetration stage. During penetration, genes encoding proteins involved in lipid and sugar metabolism are down-regulated whereas genes encoding functions controlling the transport of amino acids seemed favored. Similarly, cell wall degrading enzymes and other proteins involved in pathogenicity including RXLR effectors are highly modulated. Interestingly, *P. parasitica* uses distinct genes from these families during penetration and subsequent necrotrophic phase. We reconsidered the respective roles of the different developmental stages occurring during the infection cycle in this study. We suggest that the appressorium is involved in manipulating host cells to facilitate infection.

## Electronic supplementary material

Additional file 1: Figure S1: Pipeline used for sequence clustering, sequence origin determination and oligoarray design. (JPEG 134 KB)

Additional file 2: Table S1: Clustering of *P. parasitica* EST sequences. Sequence composition of each contig as obtained with TGICL package is presented. Each contig was named CLxContigx, with x corresponding to the cluster and contig number. (TXT 150 KB)

Additional file 3: Table S2: Functional annotation of the sequences. Predicted origin of the sequences is indicated together with BlastX result (E value < 1E-05) against the *P. parasitica* protein database (Broad Institute) and BlastX result (E value < 1E-05) against NCBI non-redundant (NR) protein database are presented together with GO annotations as proposed by blast2GO tool. (XLSX 568 KB)

Additional file 4: Table S3: Clustering of the expression patterns. Sequences contained in each expression profile clusters as obtained after K-mean clustering are presented. For all the sequences, normalized hybridization signal in each condition (mean of the two biological replicates), maximum fold change (max FC) observed between the different biological samples and BlastX best hit result (E value < 1E-05) against NCBI non-redundant (NR) protein database are indicated. For oligoarray hybridizations results, samples corresponded to mycelium grown in V8 medium (Myc), swimming zoospores (Zoo), samples enriched for appressoria differentiated on onion epidermis (App), and *A. thaliana* plantlets inoculated with *P. parasitica* zoospores and recovered at different time points of the infection : 2.5 h (appressorium-mediated penetration), 6 h (biotrophic growth, two to three cells invaded), 10 h (invasive growth along the stele) and 30 h (switch to necrotrophy). (XLSX 307 KB)

Additional file 5: Table S4: Validation of the transcriptome results and clustering analysis. mRNAs corresponding to sequences from clusters A to F were quantified by quantitative RT-PCR in samples corresponding to mycelium grown in V8 medium (Mycelium), swimming zoospores (Zoospores), samples enriched for appressoria differentiated on onion epidermis (Appressorium), and *A. thaliana* plantlets inoculated with *P. parasitica* zoospores at different time points of the infection : *A. thaliana* 2.5 hours after inoculation (appressorium-mediated penetration), *A. thaliana* 6 hours after inoculation (biotrophic growth, two to three cells invaded), *A. thaliana* 10 hours after inoculation (invasive growth along the stele), *A. thaliana* 30 hours after inoculation (switch to necrotrophy) and *A. thaliana* 4 days after inoculation (necrotrophy). Biological replicated are indicated as rep1 and rep2. Data are presented as expression ratios relative to *UBC, WS21* and Mago Nashi reference genes (2 − _CT). nd: not detected. Colum “array validation” indicates if the expression values obtained following quantitative RT-PCR show the same variation as that observed following oligoarray hybridization. Yes (except XXX) is indicated if all but one condition are in agreement with oligoarray hybridization results. Columns “cluster validation”: indicates if the observed variation corresponds to the predicted variation within each cluster (k-mean clustering, Genesis program). → Cluster X indicates if, based on the qRT-PCR expression analysis, the sequence must be analyzed with a different cluster. (XLSX 26 KB)

Additional file 6: Table S5_I: GO enrichment analysis of cluster. We estimated the differences between the observed number of sequences associated to GO terms in each cluster with expected number if randomly distributed. For each GO term, the number of sequences associated to this term in the cluster (column “occurrence in cluster”) and the number of sequences associated to this term represented on the array (column “occurrence on array”) are indicated. The total number of sequence represented in the cluster and on the oligoarray that were used for calculations of the frequencies, are mentioned (columns “sequences in cluster” and “sequences on array”). Fisher Exacts test was used to identify significant enrichment for individual GO terms. P-value of the test is indicated for each GO term. Column “sequences” indicated the names of each sequence with the corresponding GO term in the cluster. (XLSX 33 KB)

Additional file 7: Table S5_VI: GO enrichment analysis of cluster VI. (XLSX 35 KB)

Additional file 8: Table S5_III: GO enrichment analysis of cluster III. (XLSX 36 KB)

Additional file 9: Table S5_V: GO enrichment analysis of cluster V. (XLSX 22 KB)

Additional file 10: Table S5_II: GO enrichment analysis of cluster II. (XLSX 40 KB)

Additional file 11: Table S5_IV: GO enrichment analysis of cluster IV. (XLSX 83 KB)
