# Supplementary figures and images for: Transcriptome dynamics of Arabidopsis thaliana root penetration by the oomycete pathogen Phytophthora parasitica
Source: BMC Genomics. 2014 Jun 29;15(1):538. doi: 10.1186/1471-2164-15-538 (PMC4111850; doi:10.1186/1471-2164-15-538)

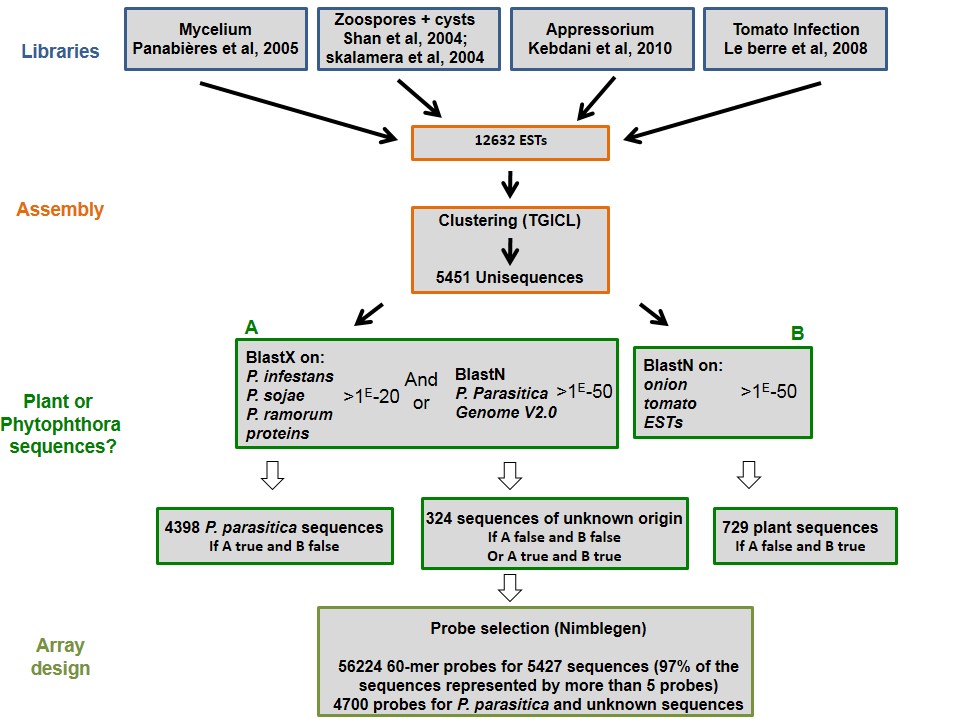

Supplement: Supplementary file 1 — Additional file 1: Figure S1: Pipeline used for sequence clustering, sequence origin determination and oligoarray design. (JPEG 134 KB) [file 12864_2013_6272_MOESM1_ESM.jpeg]
